# Experimental Study on the Laser Transmission Joining of Polystyrene and Titanium

**DOI:** 10.3390/ma11091513

**Published:** 2018-08-23

**Authors:** Pin Li, Jing Li, Wensheng Tan, Huixia Liu, Xiao Wang

**Affiliations:** 1 School of Mechanical Engineering, Shanghai Jiao Tong University, Shanghai 200000, China; 2 School of Mechanical Engineering, Jiangsu University, Zhenjiang 212013, China; lip@ujs.edu.cn (P.L.); 17315764980@163.com (J.L.); wx@ujs.edu.cn (X.W.); 3 Changzhou Key Laboratory of Large Plastic Parts Intelligence Manufacturing, Changzhou College of Information Technology, Changzhou 213164, China; tws.163@163.com

**Keywords:** laser transmission joining, laser surface treatment, oxygen plasma treatment, contact angle, surface free energy, XPS, chemical bonds

## Abstract

To address the difficulty of joining polystyrene (PS) and titanium by laser transmission joining, two methods—laser treatment of the titanium surface and oxygen plasma treatment of the PS surface—are used to compare the laser transmission joint strengths of the different treatment methods. The results of the experiments find that joining with titanium can be achieved only when PS is treated with oxygen plasma. When the laser-treated surface of titanium is jointed to the oxygen plasma-treated PS, the joint strength is the highest, reaching 6.5 MPa. The joining mechanism of oxygen plasma-treated PS and laser oxidation-treated titanium was investigated by joint tensile failure mode, joint micromorphology observation, contact angle and surface free energy experiments, and X-ray photoelectron spectroscopy (XPS). The results show that the failure mode of the joint is an interfacial failure; the size and amount of bubbles play an important role in the joining strength, and the joints with fine and uniform bubbles have the highest joint strength. The two surface treatment methods can improve the surface energy of the joints, improve the compatibility between the two joining surfaces, and enhance the joint strength. Ti–C and Ti–O chemical bonds are formed at the joints, which are the main reason for the increase in joint strength.

## 1. Introduction

Due to the difference in the mechanical and physical properties between the metals and polymers, joining a metal and a polymer is often difficult, and the combined behaviors are not yet fully understood. Mechanical fastening and adhesive bonding are commonly applied in joining a metal and a polymer; however, these methods have some drawbacks, such as low joining efficiency and a high environmental impact [1]. New joining processes have been developed recently involving laser joining, ultrasonic joining, and friction-assisted joining to exploit the possibility of obtaining high-quality joints of metals and polymers [2,3,4]. These methods may play an important role in different applications; however, ultrasonic joining is limited to small components with joining lengths typically not exceeding a few centimeters; and friction-assisted joining requires very stiff clamping system due to high loads involved during the processes. Laser transmission joining has become a joining process for metals and polymers, and is widely used in a variety of industrial areas including automobiles, aircraft, and electronics [5,6,7]. This is because of advantages such as good joining quality, small deformation, high efficiency, easy operation, and non-contact interaction. In the past few years, Mian conducted the joining experiment of polyimide (PI) and titanium-coated glass, and analyzed the joining mechanism [8]. Song et al. studied the laser transmission joining of polyethylene terephthalate (PET) and 316L stainless steel, and revealed the change rule for joint quality with respect to changes in the process parameters [9]. Georgiev determined that the reason a strong joint was formed between Teflon^TM^ (fluorinated ethylene propylene (FEP), Wilmington, DE, USA) and Ti was the formation of Ti–F chemical bonds at the joint [10]. Wang et al. studied the joint of PET and Ti, and found a formation of Ti–C chemical bonds between PET and Ti [11,12].

To improve the joint strength, the metal surface can be anodized or microtextured; e.g., Roesner et al. studied the laser transmission joining of various polymers such as glass fiber- reinforced nylon 66, nylon 66, and polycarbonate with metal, and processed the groove on the metal surface by a laser ablation method. During the joining process, the upper layer of molten polymer flowed into the groove to form a mechanical riveting, thereby increasing the joint strength [13]. Bergmann et al. studied the joining between nylon and a steel plate, and effectively improved the joint strength by producing a relatively rough surface on the steel plate through manual sandblasting, and then performing the laser transmission joining [14]. Lambiase et al. conducted laser sculpturing on the aluminum substrate to produce teeth features, and then investigated the laser-assisted joining of aluminum alloy sheets with polyvinyl chloride (PVC) and polyetheretherketone (PEEK) [5,15]. Yusof et al. investigated the joining of PET and surface-anodized A5052. From the experiment, the authors found that both PET and anodized A5052 and PET and untreated A5052 clearly formed molten pools, and the joint strength was mainly affected by the heat input, rather than the pulse duration. At the same time, the generation of bubbles and the depth of the molten pool were two aspects that affected the joint strength [16]. Zhang et al. investigated the joining of aluminum alloy A6061 and carbon fiber-reinforced plastic (CFRP). A phosphate-anodizing pretreatment of A6061 was carried out [17]. Jung et al. conducted a pre-oxidation process on the surface of the zinc-coated steel using a selected temperature and air atmosphere in a furnace. A thick zinc oxide layer that reacted on the zinc-coated steel increases the possibility of chemical bonding between zinc oxide and carbon (Zn–O–C) [7]. In summary, the pretreatment methods for improving the strength of the joint are mainly through the microtexturing of the metal or the anodization of the metal surface, while research on the treatment of the polymer surface is relatively less. Arai et al. studied the laser transmission joining of cyclic olefin polymers (COP) and SUS304 through surface UV-ozone treatments, and found that the increase in the surface energy of COP improved the surface interaction between COP and SUS304, and thus improved the joint strength [18].

Polystyrene (PS) and titanium are widely used in the biomedical applications. PS is one of the important polymers in the numerous applications for the fabrication of everyday life objects, and has been widely used in biomedical material as tissue culture dishes for its aging resistance, high transparency, low toxicity, good rigidity, etc. The transmission joining between PS and a thin titanium plate shows that the joint strength of untreated PS and titanium is very low. PS is a non-polar material. Its molecular chain does not contain polar groups, so the surface energy is very low. Using the plasma treatment can generate oxygen-containing functional groups on the PS surface. In different plasma atmospheres (H_2_, Ar, N_2_, air, or O_2_), PS gains various functional groups. Oxygen plasma-treated PS has a relatively high percentage of oxygen atoms and a high compositional ratio of O/C [19]. 

In this paper, based on the above methods for the surface modification of polymers and the surface treatment of metals, it is proposed to first perform an oxygen plasma treatment of PS and a laser surface treatment of titanium, and then perform a laser transmission joining of PS and titanium plates. This paper focuses on the strength of joints obtained by four different treatment methods, and investigated the joining mechanism of PS and titanium by tensile failure mode, joint micromorphology observation, contact angle and surface free energy experiments, and X-ray photoelectron spectroscopy (XPS) analysis.

## 2. Experimental Procedures

### 2.1. Experimental Equipment and Sample Preparation

Laser transmission joining and laser surface treatment were conducted with a semiconductor laser (Compact 130/140, DILAS Diodenlaser GmbH, Mainz, Germany) with an output wavelength of 980 ± 10 nm, a minimum spot diameter of 700–800 μm with a circular shape, a maximum output power of 130 W, and a working temperature kept between 15–25 °C. 

A universal tensile machine (UTM4104, Shenzhen Suns Technology Co., Ltd., Shenzhen, China) was used for testing the joint strength, with v = 2 mm/min. The lap shearing test finally broke the joints through loading tension at both ends of the joints. In this paper, the joint strength is measured by shear stress, and the shear stress is calculated as Formula (1):

(1) $$\;\sigma =\frac{F}{W\times L\;}$$

An ultra-depth optical microscopy (VHX-1000, Keyence Corporation, Osaka, Japan) was used to observe the micromorphology of the joint. 

Contact angle measurements (OCA40, Dataphysics Instruments GmbH, Stuttgart, Germany) were performed to study the changes in the contact angles of the surface. 

An X-ray photoelectron spectrometer (ESCALAB 250Xi, Thermo Fisher Scientific, Waltham, MA, USA) was used for the interface analysis of the chemical composition and chemical bond state before and after treatments.

The dimensions of all of the samples were 150 mm × 20 mm × 0.1 mm. The main properties of the materials are shown in Table 1. Before the experiments, the titanium and PS surfaces were cleaned using an ultrasonic cleaning machine with ethanol to remove impurities, and drying in a dry machine for 12 h.

### 2.2. Experimental Methods

#### 2.2.1. Laser Surface Treatment

**Figure 1:**
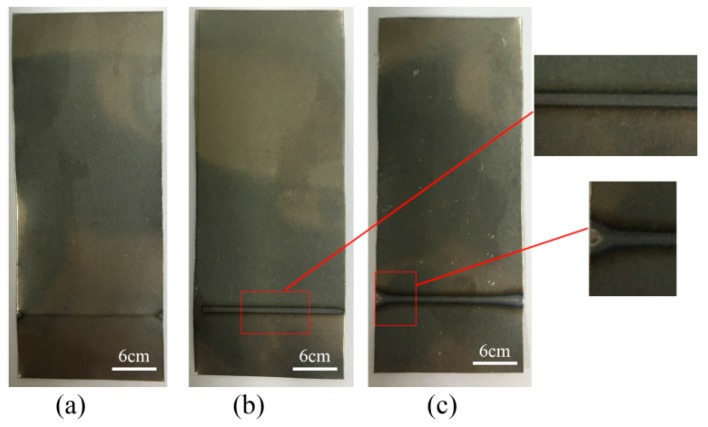
The samples of the laser-treated titanium by the laser power for surface treatment. (**a**) 4 W; (**b**) 5 W; (**c**) 6 W.

The essence of the laser surface treatment includes scanning the titanium metal surface by the laser in the air atmosphere, oxidizing by heating, and forming an oxide layer of a certain thickness on the metal surface [20]. Figure 1 shows the samples of the titanium surface treated by the laser power for surface treatment of 4 W, 5 W, and 6 W. The scanning speed was 2 mm/s, the number of scans was six, and the spot diameter was 2 mm. From Figure 1, oxidation is not apparent at the laser power of 4 W, because of the low laser power for surface treatment. When the laser power is 5 W, an oxide layer of uniform width is produced along the scan direction of the laser, and the titanium surface oxidization is optimal. When the laser power is 6 W, the surface temperature of the titanium is high, causing the melt of the titanium surface. 

The topography of titanium after the laser treatment could be altered, and could promote the joint strength by a mechanical interference effect. In this study, which focuses on what is attributed to the chemical bond between titanium and the polymer, the process parameters for laser surface treatment are fixed and shown in Table 2. 

#### 2.2.2. Oxygen Plasma Treatment

Plasma treatment is an effective method for the surface modification of polymers [20]. In this study, plasma treatment apparatus (HD-1B, Changzhou Zhongke Changtsi Plasma Processing Apparatus Plasma Technology Co., Ltd., Changzhou, China) was used. The samples to be treated were placed in the instrument’s reaction chamber made of hard and high temperature-resistant glass. For the plasma pretreatment, the output power of 150 W was used with the working gas of oxygen and the pressure of 40 Pa. The pretreatment time was 120 s. The materials after plasma pretreatment were placed for 90 min, and then, the laser transmission welding experiments were carried out. 

#### 2.2.3. Laser Transmission Joining

**Figure 2:**
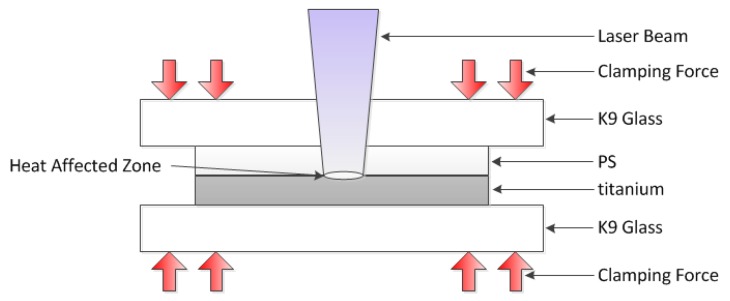
Schematic of laser transmission joining between PS and titanium.

For the laser transmission joining experiment, the PS sample was used as the upper transparent material, and the thin titanium was the lower material. The titanium surface was selected as the laser absorber. Figure 2 presents a schematic of laser transmission joining between PS and titanium. The experiment was performed in the form of the lap joint, using the K9 glass as the clamping layer, and a certain clamping pressure of 0.5 MPa was applied. The process parameters and limits for laser joining are shown in Table 3. Three replicates were performed for each test condition.

## 3. Results and Discussion

### 3.1. Joint Strength of Different Treatment Methods

**Figure 3:**
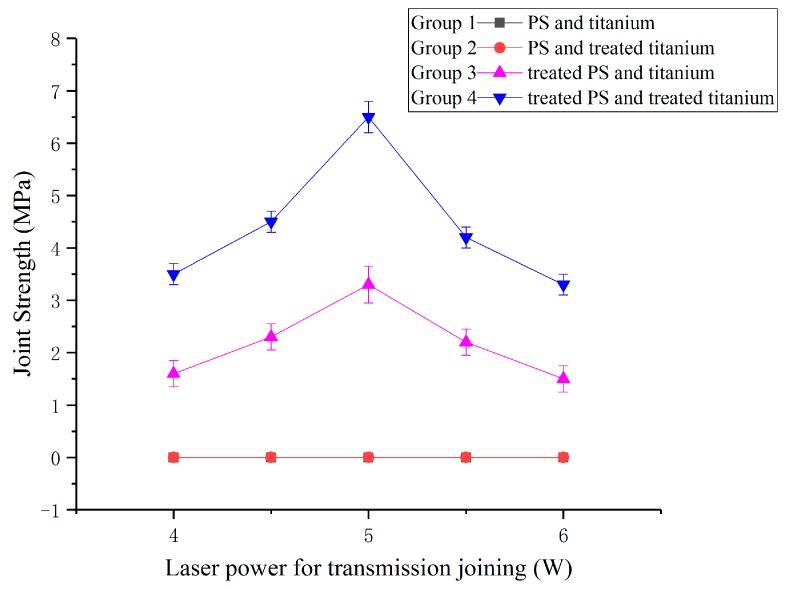
The influence of treatments and laser power for joining on the joint strength.

This paper includes four groups of contrasting experiments: The first group consists of PS and titanium; the second group consists of PS and laser-oxidized titanium; the third group consists of oxygen plasma-treated PS and titanium; and the fourth group consists of oxygen plasma-treated PS and laser-oxidized titanium. The experimental result of each combination is shown in Figure 3. From the graph, the joint strengths of the first and second groups are 0 MPa, the maximum joint strength of the third group is approximately 3 MPa, and the maximum joint strength of the fourth group is 6.5 MPa.

**Figure 4:**
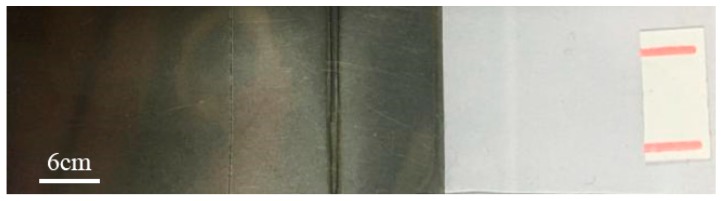
The joining sample of oxygen plasma-treated PS and laser-oxidized titanium.

PS without oxygen plasma treatment cannot be joined with titanium. However, oxygen plasma-treated PS achieves joining with titanium under the same process parameters. In addition, the maximum joint strength of oxidized titanium and treated PS is approximately two times that of unoxidized titanium and treated PS. Therefore, oxygen plasma-treated PS is a necessary condition to accomplish the joining, and the use of laser-oxidized titanium can improve the joint strength. Figure 4 shows the sample of transmission joining between an oxygen plasma-treated PS and a laser-oxidized titanium plate.

### 3.2. Tensile Failure Analysis

**Figure 5:**
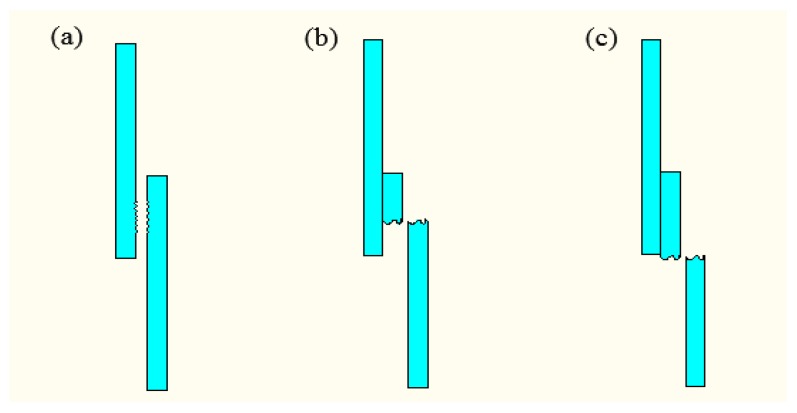
Tensile failure modes. (**a**) Interface failure; (**b**) The base fracture along the joint; (**c**) The base fracture in the middle.

As shown in Figure 5, three tensile failure modes are evident: failure at the joint interface, the base fracture along the joint, and the base fracture in the joint in the middle. When the joint strength is smaller than the strengths of the based materials, failure occurs at the joint interface. While the joint strength is higher, the base fracture occurs along the joint or even in the middle.

**Figure 6:**
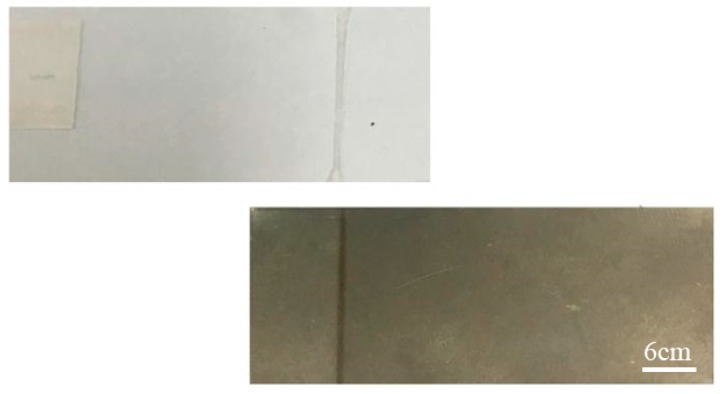
The sample of oxygen plasma-treated PS and laser-oxidized titanium after tensile test.

The tensile failure mode of the joint between oxygen plasma-treated PS and oxidized titanium is interfacial failure, as shown in Figure 6. The joint strength is less than the strengths of PS and titanium, and the failure occurs at the joint interface.

### 3.3. Joint Micromorphology Observation

**Figure 7:**
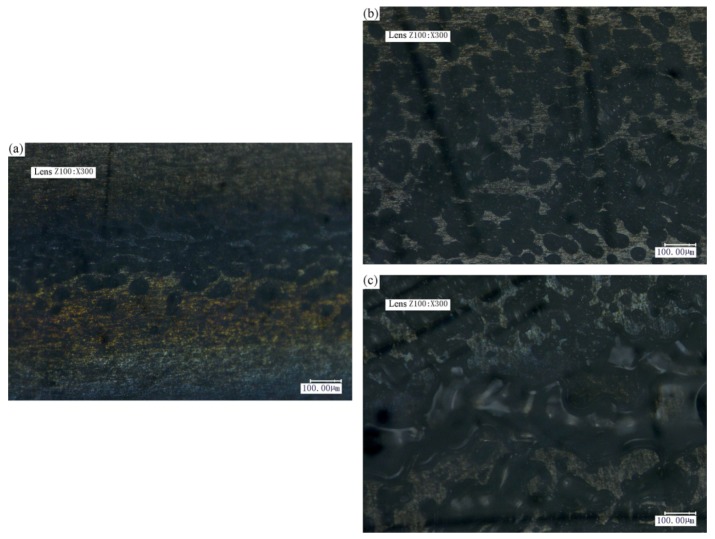
Micromorphology of the joints by laser power for transmission joining. (**a**) 4 W; (**b**) 5 W; (**c**) 6 W.

By observing the micromorphology using an ultra-deep optical microscope, generated bubbles were found at the joint. Figure 7 presents the micromorphologies of the joints by laser powers for the transmission joining of 4 W, 5 W, and 6 W.

Figure 7a shows that the number of bubbles at the joint is little, and the bubble size is small, when the laser power for joining is 4 W. This result indicates that this laser power for joining is too weak, and the PS is insufficiently heated, with few bubbles being seen.

Figure 7b is the micromorphology of the joint when the laser power for joining is 5 W. The number of bubbles has significantly increased. This result shows that the PS is sufficiently heated, fine, and generating uniform bubbles. 

Figure 7c reveals the micromorphology of the joint when the laser power for joining is 6 W. The number of bubbles is decreased, and the bubble size becomes larger. This is because the laser power for joining is too high; the evaporation of the polymer is promoted due to the higher temperatures in the joint interface. Due to this higher temperature, the diffusion time is also longer; therefore, the coalescence of the bubbles is promoted.

The result indicates that the size and amount of bubbles play an important role in the joining strength. As shown in Figure 3, the joint strength is approximately 3.5 ± 0.2 MPa when the laser power for joining is 4 W, approximately 6.5 ± 0.3 MPa when the laser power for joining is 5 W, and approximately 3.3 ± 0.2 MPa when the laser power for joining is 6 W. It can be concluded that the joint with fine and uniform bubbles exhibits the highest joint strength.

Many scholars have also demonstrated that the size and amount of bubbles would play an important role on the joining strength. Liu et al. studied the joining of an aluminum alloy and PET, and found that the bubbles induced high pressure into molten polymer, and then this molten polymer was pushed onto the metal surface [21]. Katayama et al. have elaborated on how the formation of bubbles could improve the joint strength between polymers and metals [22]. Farazila et al. posited that a large amount and coarser bubbles may reduce the joining strength if the bubbles play as defects [23].

### 3.4. Contact Angle and Surface Energy

(1) Surface energy formulas

Contact angles can be used to characterize the wettability of a solid surface [24]. When the water contact angle of a polymer surface is equal to or greater than 80°, the material is hydrophobic, and the surface does not present polar groups. Materials with a high wettability also possess high surface tensions [25].

The calculation for the surface tensions (γ) is given by Formula (2) [26]:

(2) $$\;\gamma =\gamma^{D}+\gamma^{P}\;$$

In Formula (1), γ*^D^* represents the non-polar portion of the surface tensions, and γ*^p^* represents the polar portion of the surface tensions. The non-polar part comes from the Van der Waals interaction force; on the other hand, the polar part was the geometric mean of the corresponding acid and alkali. Since the contact angle between a liquid and solid is determined by both the surface tension of the liquid and the surface free energy of the solid, the surface free energy of the solid can be calculated via at least two kinds of liquid surface tension with their surface contact angle. The polar and non-polar portions of the surface tensions of a material can be calculated by Formulas (3) and (4):

(3) $$\;\gamma_{l1\;}\left(1+\cos\theta_{1}\right)=2\left[{\left(\gamma_{l1}^{D}\gamma^{D}\right)}^{0.5}+{\left(\gamma_{l1}^{P}\gamma^{P}\right)}^{0.5}\right]$$

(4) $$\;\gamma_{l2\;}\left(1+\cos\theta_{2}\right)=2\left[{\left(\gamma_{l2}^{D}\gamma^{D}\right)}^{0.5}+{\left(\gamma_{l2}^{P}\gamma^{P}\right)}^{0.5}\right]$$

In Formulas (3) and (4), $\gamma_{l1}$ and $\gamma_{l2}$ represent the total surface tensions of the two liquids, respectively; *θ*_1_ and *θ*_2_ represent the angles between two types of liquid; $\gamma_{l1}^{D},\;\;\gamma_{l2}^{D}$ and $\gamma_{l1}^{P},\;\;\gamma_{l2}^{P}$ represent the non-polar and polar contributions of the surface tension of the two liquids, respectively; and $\gamma_{s}^{D}$ and $\gamma_{s}^{P}$ represent the non-polar and polar portions of the surface free energy of the solid.

(2) Contact angle tests

Contact angle tests are used to quantitatively analyze the contact angle and surface energy changes of the treated PS and laser-treated titanium. Two known liquids, pure water and ethylene glycol, were selected for the contact angle tests. Table 4 provides the surface tension of pure water and ethylene glycol.

**Figure 8:**
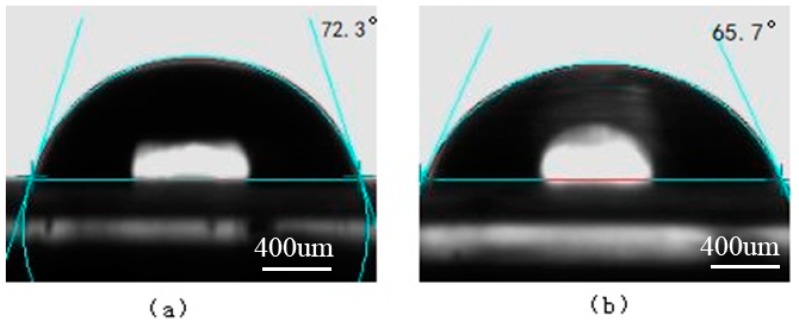
The surface contact angles on the PS surface. (**a**) Water; (**b**) Ethylene glycol.

**Figure 9:**
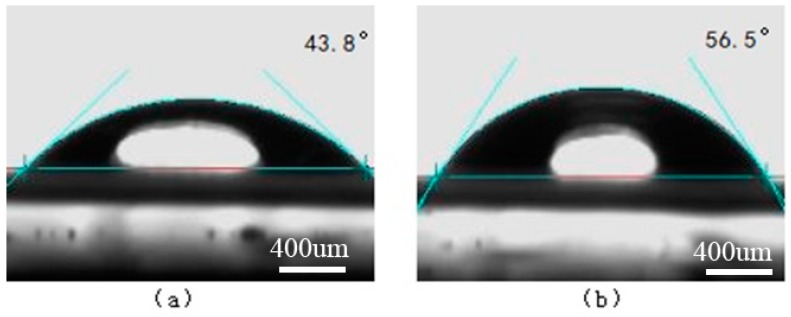
The surface contact angles on the oxygen plasma-treated PS surface. (**a**) Water; (**b**) Ethylene glycol.

**Figure 10:**
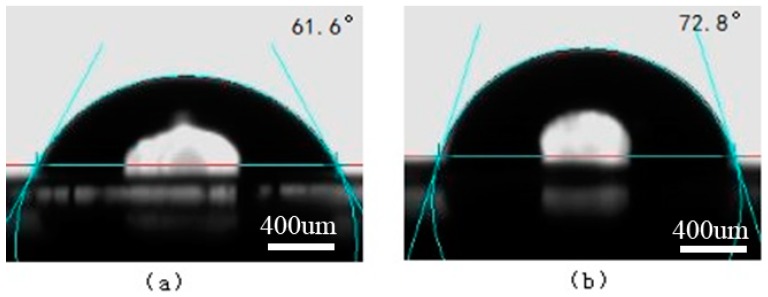
The surface contact angles on the Ti surface. (**a**) Water; (**b**) Ethylene glycol.

**Figure 11:**
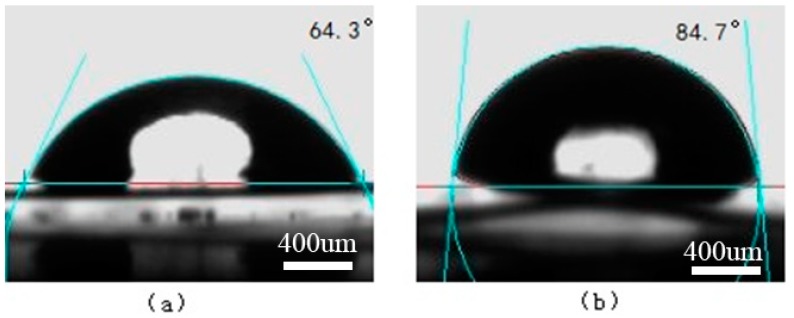
The surface contact angles on the laser-treated Ti surface. (**a**) Water; (**b**) Ethylene glycol.

The surface contact angles of water and ethylene glycol on the PS and oxygen plasma-treated PS surfaces are shown in Figure 8 and Figure 9, respectively. The surface contact angles of water and ethylene glycol on the Ti and laser-treated Ti surfaces are shown in Figure 10 and Figure 11, respectively.

By the surface tensions formula, the surface tensions of each experimental material can be calculated, and the results are given in Table 5.

As shown in Table 5, the surface tensions of oxygen plasma-treated PS increased a lot, and the surface tensions of the laser-treated titanium similarly improved. Therefore, it is concluded that the oxygen plasma treatment and the laser surface treatment methods can improve the surface tensions of the joint materials, improve the compatibility between the oxygen plasma-treated PS and the laser-treated titanium, raise the surface chemical activity and generate new chemical bonds, and contribute to the joint strength enhancement.

Many scholars have demonstrated that an increase in surface energy can improve joint strength. Arai et al. compared the surface energy of a COP surface before and after a UV-ozone treatment and found that the increase in the surface energy of COP improved the surface interaction between COP and SUS304, and thus improved the joint strength [18].

### 3.5. XPS Analysis

**Figure 12:**
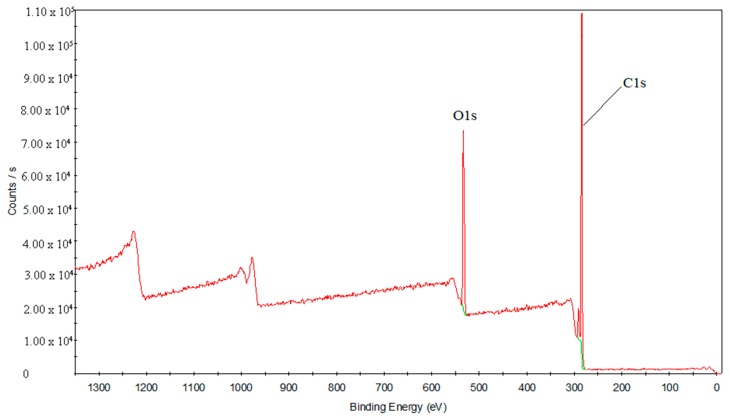
The element’s full spectrum diagram of the PS surface.

**Figure 13:**
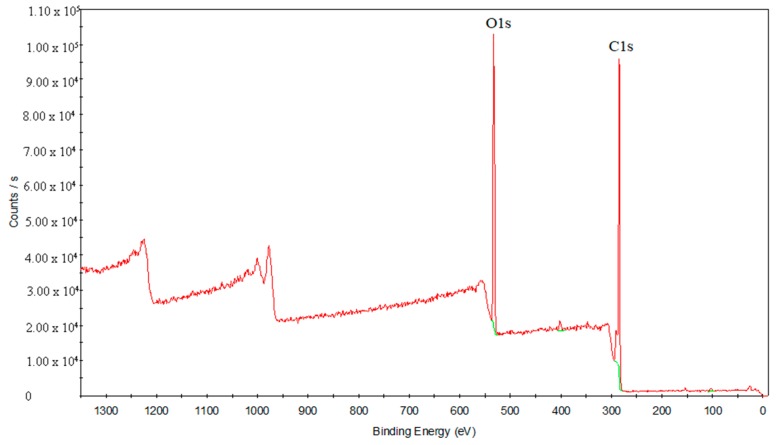
The element’s full spectrum diagram of the oxygen plasma-treated PS surface.

To study the laser transmission joining mechanism in depth, XPS analysis was used to study the interface analysis of the chemical composition and chemical bond state before and after treatments. Figure 12 presents the elements’ full spectrum diagram of the PS surface. C and O elements are detected in this area. Figure 13 shows the element’s full spectrum diagram of the oxygen plasma-treated PS surface. The results show that the O element significantly increases after the treatment with oxygen plasma, even exceeding the content of the C element. 

**Figure 14:**
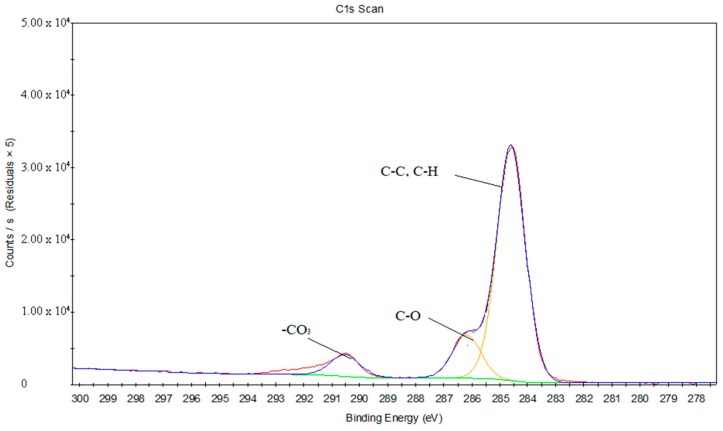
The C1s peak fits of the PS surface.

**Figure 15:**
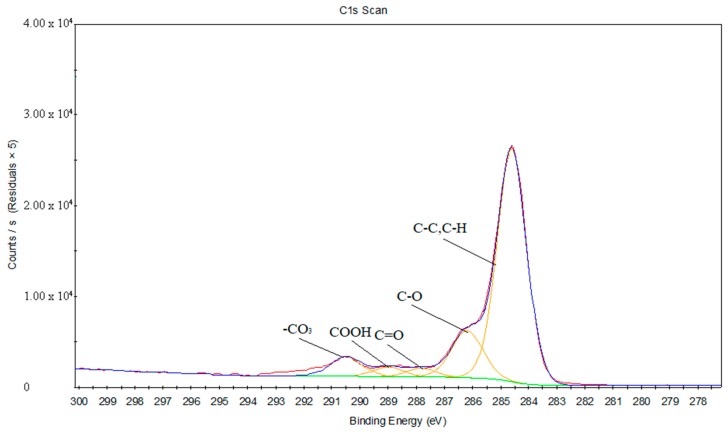
The C1s peak fits of the PS surface treated by oxygen plasma.

Figure 14 and Figure 15 reveal the C1s peak fits of the PS and treated PS, respectively. Table 6 and Table 7 provide the chemical bond information corresponding to the C1s peak fits. It can be inferred that C=O and COOH groups are existing on the PS surface after the oxygen plasma treatment, and appearing around 287.82 eV and 288.99 eV, respectively.

**Figure 16:**
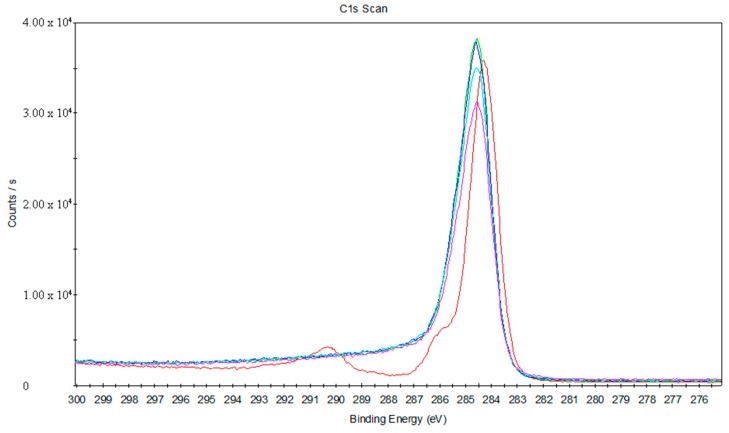
The C1s peak fits of the joint between the treated PS and titanium.

Figure 16 shows the C1s peak fits of the joint between the treated PS and titanium. Ar^+^ ions were used to sputter the stripped joint for different lengths of time (0 s, 25 s, 45 s, 120 s, and 360 s). Ramqvist et al. reported that a peak around 281.7eV in the C1s peak fits represented the existence of a Ti–C chemical bond [27]. Therefore, it can be inferred that there is no formation of Ti–C chemical bonds at the joint between the treated PS and titanium.

**Figure 17:**
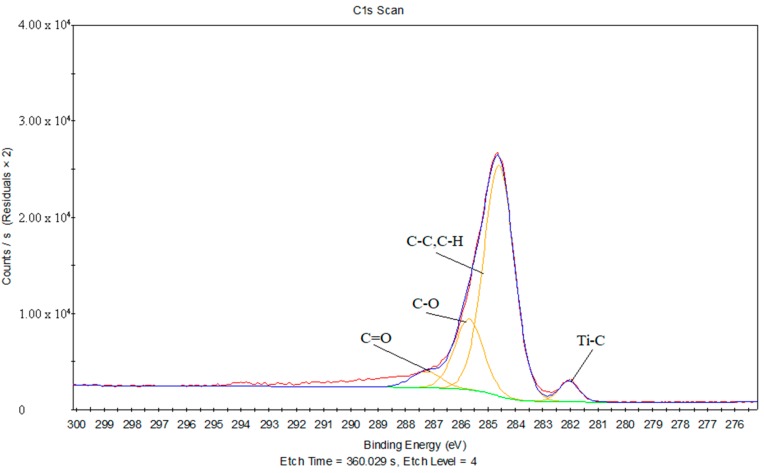
The C1s peak fits of the joint between the treated PS and laser-oxidized titanium.

Figure 17 provides the C1s peak fits of the joint between the treated PS and laser-oxidized titanium, and Table 8 gives the chemical bonds information corresponding to the C1s peak fits of the joint between the treated PS and laser-oxidized titanium. It can be inferred that the peaks are 282.05 eV, which is close to 281.7 eV, and is likely the Ti–C chemical bond. Due to the combination of C–C and C–H contributions, a peak at 284.6 eV is generated. Furthermore, the peaks at 285.69 eV and 287.25 eV correspond to C–O and C=O, respectively. 

**Figure 18:**
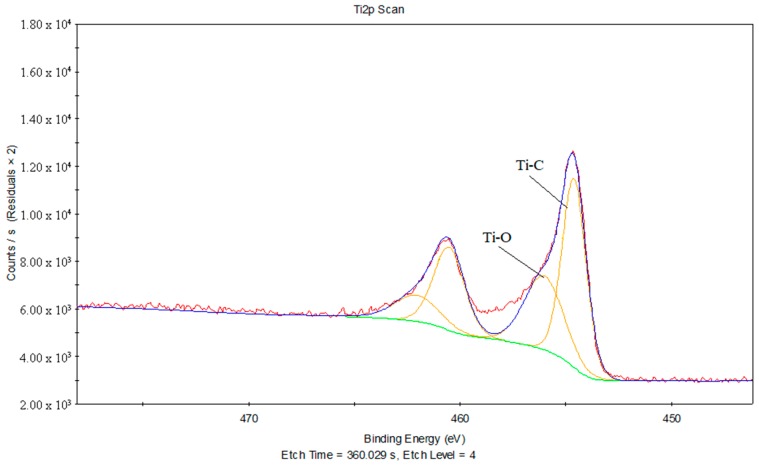
The Ti2p peak fits of the joint between the treated PS and laser-oxidized titanium.

Figure 18 shows the Ti2p peak fits of the joint between the treated PS and laser-oxidized titanium, and Table 9 provides the chemical bonds information corresponding to the Ti2p peak fits of the joint between the treated PS and laser-oxidized titanium. It can be inferred that there is a Ti–C chemical bond around 454.61 eV, and a Ti–O chemical bond around 456.03 eV.

Above all, in the process of laser transmission of PS and titanium, it is concluded that no formation of Ti–C chemical bonds occur at the joint between the treated PS and titanium, while Ti–C and Ti–O forms exist at the joint between the treated PS and laser-oxidized titanium. It is evident that the formation of Ti–C and Ti–O chemical bonds is one of the fundamental reasons that the joint strength is the highest for the oxygen plasma-treated PS and laser-oxidized titanium.

Some scholars have proved that the joint strength is high for a joint accompanied by the formation of chemical bonds. Wang et al. studied the laser transmission joining of a biocompatible PET thin film and titanium thin plate. They investigated the joining mechanism of the substrates using XPS analysis, and believed that the reason for the formation of the high-strength joint was the existence of the Ti–C chemical bond [7]. Georgiev et al. studied a PI and titanium thin film joint, and found the formation of chemical bonds at the interface by analytical techniques. Their study found that the presence of chemical bonds was the main reason for the high joint strength [28].

## 4. Conclusions

This work presents the laser transmission joining experiments of PS and titanium, the introduction of two pretreatment methods (laser surface treatment and oxygen plasma surface treatment), and a comparison of the joint strength using the different treatment methods. Finally, after an investigation of the joining mechanism by analysis of the fracture mode, contact angle and surface free energy experiments, and XPS analysis, the following conclusions are obtained:

(1) The oxygen plasma-treated PS is a necessary condition to achieve the joining of PS and titanium. The joint strength is the highest when the laser-treated surface of titanium is joined to the oxygen plasma-treated PS.

(2) The tensile failure mode of the laser transmission joining of the oxygen plasma-treated PS and oxidization-treated titanium is interfacial failure.

(3) The size and amount of bubbles play an important role in the joining strength, and the joints with fine and uniform bubbles have the highest joint strength.

(4) The oxygen plasma treatment and laser oxidization treatment can increase the surface energies of the joint materials, improve the compatibility between the oxygen plasma-treated PS and the laser-treated titanium, raise the surface chemical activity, generate new chemical bonds, and contribute to the joint strength enhancement.

(5) Ti–C and Ti–O forms exist at the joint between the treated PS and laser-oxidized titanium. The formation of Ti–C and Ti–O chemical bonds is the main reason that the joint strength is the highest for the oxygen plasma-treated PS and laser-oxidized titanium.

## Figures and Tables

**Table 1:** Main properties of titanium and polystyrene (PS).

Property	Titanium	PS
Density (g/cm^3^)	4.54	1.05
Specific heat J/(kg·K)	520	1300
Thermal conductivity (W/mK)	15.24	0.08
Melting temperature, Tm (°C)	1668	240
Ultimate tensile stress (MPa)	539	60

**Table 2:** Process parameters and limits for laser surface treatment.

Parameter	Limits
Laser power for surface treatment (W)	5
Laser scanning speed (mm/s)	2
Number of scans (n)	6
Spot diameter (mm)	2

**Table 3:** Process parameters and limits for laser joining.

Parameter	Limits				
Laser power (W)	4	4.5	5	5.5	6
Laser scanning speed (mm/s)	2	2	2	2	2
Defocus distance (mm)	1	1	1	1	1

**Table 4:** The surface tension of pure water and ethylene glycol.

Liquid Type	$\gamma_{l}\;\mathrm{mN}/m$	$\gamma^{D}\;\mathrm{mN}/m$	$\gamma^{P}\;\mathrm{mN}/m$
Pure water	75 ± 0.5	21.6 ± 0.5	53.4 ± 0.5
Ethylene glycol	48 ± 0.5	29 ± 0.5	19 ± 0.5

**Table 5:** The surface tensions of the experimental materials.

Material Type	γ	$\gamma^{D}\;\mathrm{mN}/m$	$\gamma^{P}\;\mathrm{mN}/m$
PS	34.01	3.25	30.76
Oxygen plasma-treated PS	83.85	0.24	83.61
Ti	65.13	0.53	64.6
Laser-treated Ti	82.13	5.02	77.11

**Table 6:** The chemical bonds information corresponding to the C1s peak fits of the PS surface.

Binding Energy (eV)	Full Width at Half Maximum (FWHM)	Chemical Bond State
284.6	1.18	C–C, C–H
286.19	1.17	C–O
290.51	1.23	–CO_3_

**Table 7:** The chemical bonds information corresponding to the C1s peak fits of the PS surface treated by oxygen plasma.

Binding Energy (eV)	FWHM	Chemical Bond State
284.6	1.22	C–C, C–H
286.19	1.3	C–O
287.82	1.16	C=O
288.99	1.21	COOH
290.48	1.29	–CO_3_

**Table 8:** The chemical bonds information corresponding to the C1s peak fits of the joint between the treated PS and laser-oxidized titanium.

Binding Energy (eV)	FWHM	Chemical Bond State
282.05	0.86	Ti–C
284.6	1.3	C–C, C–H
285.69	1.22	C–O
287.25	1.3	C=O

**Table 9:** The chemical bonds information corresponding to the Ti2p peak fits of the joint between the treated PS and laser-oxidized titanium.

Binding Energy (eV)	FWHM	Chemical Bond State
454.61	1.33	Ti–C
456.03	2.18	Ti–O
